# Archaeological Evidence for Peach (*Prunus persica*) Cultivation and Domestication in China

**DOI:** 10.1371/journal.pone.0106595

**Published:** 2014-09-05

**Authors:** Yunfei Zheng, Gary W. Crawford, Xugao Chen

**Affiliations:** 1 Zhejiang Provincial Institute of Relics and Archaeology, Hangzhou, China; 2 Department of Anthropology, University of Toronto Mississauga, Mississauga, Ontario, Canada; Chinese Academy of Sciences, China

## Abstract

The cultivated/domesticated peach (*Prunus persica* var. *persica*; Rosaceae, subgenus *Amygdalus*; synonym: *Amygdalus persica*) originated in China, but its wild ancestor, as well as where, when, and under what circumstances the peach was domesticated, is poorly known. Five populations of archaeological peach stones recovered from Zhejiang Province, China, document peach use and evolution beginning ca. 8000 BP. The majority of the archaeological sites from which the earliest peach stones have been recovered are from the Yangzi River valley, indicating that this is where early selection for favorable peach varieties likely took place. Furthermore, peach stone morphology through time is consistent with the hypothesis that an unknown wild *P. persica* was the ancestor of the cultivated peach. The oldest archaeological peach stones are from the Kuahuqiao (8000–7000 BP) and Tianluoshan (7000–6500 BP) sites and both stone samples segregate into two size groups, suggesting early selection of preferred types. The first peach stones in China most similar to modern cultivated forms are from the Liangzhu culture (ca. 5300 to 4300 BP), where the peach stones are significantly larger and more compressed than earlier stones. Similar peach stones are reported from Japan much earlier (6700–6400 BP). This large, compressed-stone peach was introduced to Japan and indicates a yet unidentified source population in China that was similar to the Liangzhu culture peach. This study proposes that the lower Yangzi River valley is a region, if not *the* region, of early peach selection and domestication and that the process began at least 7500 years ago.

## Introduction

**Figure 1:**
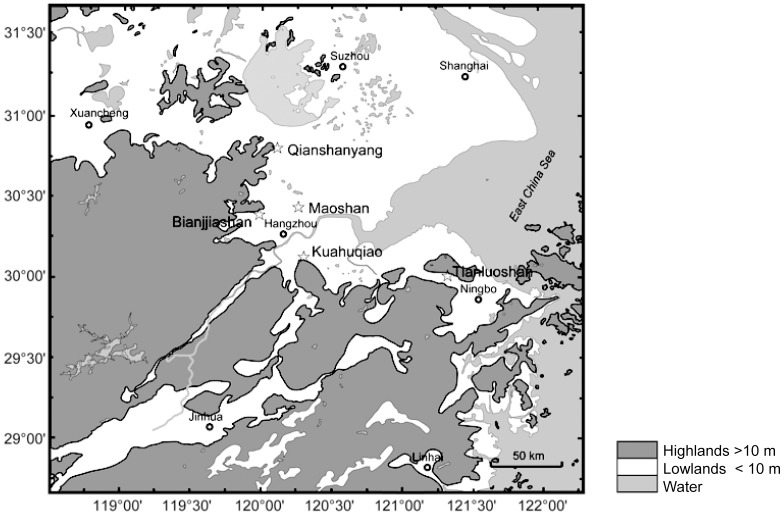
Location of the Kuahuqiao, Tianluoshan, Maoshan, Bianjiashan, and Qianshanyang sites.

The domestication of perennial plants, most of which (about 75%) are propagated by cloning, has received limited attention compared to annual plants [1]–[4]. Perennial fruit domestication mainly involves long-lived woody taxa that produce edible fruit [2]. A wide variety of trees and shrubs developed significant economic importance in China: apricot (*Prunus armeniaca*), chestnut (*Castanea* spp.), Chinese bayberry (*Myrica rubra*), hawthorn (*Crataegus* spp.), hazelnut (*Corylus* spp.), jujube (*Ziziphus jujube*), litchi (*Litchi chinensis*), manadarin orange (*Citrus reticulata*), paper mulberry (*Broussonetia papyrifera*), peach (*Prunus persica*) and tea (*Camellia chinense*) [5]. Their management and/or domestication have received little attention from an archaeological perspective. The focus on rice (*Oryza sativa*) domestication in the Yangzi valley, although important, needs to be balanced against a more holistic examination of other organisms that became components of agricultural systems in the region [6]. A significant sample of peach remains has been recovered from over 24 archaeological sites, most in the lower Yangzi valley, and two Jomon sites in Kyushu, Japan in contexts dating between 8000 and 2200 cal. BP (Fig. 1, Fig. S1, Table S1; all BP dates are calibrated unless otherwise indicated). Peach stones are not reported from Korea during this period, the oldest peach stones so far recovered being from the Three Kingdoms Period (AD 57–668; note: AD and BC are used for historically dated periods). This paper presents the first detailed quantitative and qualitative examination of tree fruit domestication in China by providing a comparative analysis of archaeological peach stones from five sites in the lower Yangzi valley and examines when, where, and under what circumstances peach began its close relationship with people. In order to examine these questions, we first address the visibility of the evolution of the peach domestication syndrome in the archaeological record. The ancestry of peach is also examined in relation to the closely related taxa that grow in China today. Early plant management and selection in the Yangzi valley not only included rice but at least one arboreal taxon too, the peach.

## Materials and Methods

**Figure 2:**
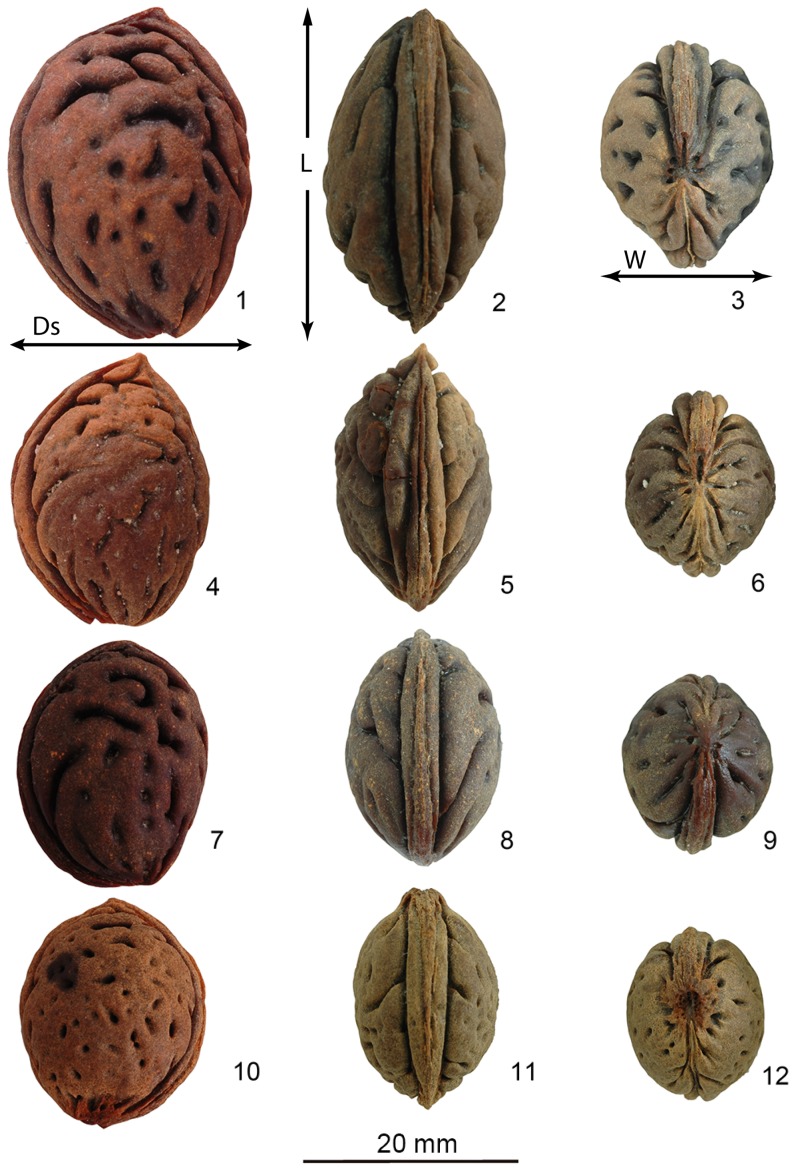
Archaeological remains of peaches. 1, 2, 3, Qianshanyang site; 4, 5, 6, Maoshan site; 7–12, Kuahuqiao illustrating two variants of peach stones, one with short grooves and small pits and the other with prominent grooves and pits. L = length, W = width and Ds = suture diameter.

Peach stones recently excavated from the Kuahuqiao, Tianluoshan, Bianjiashan, Maoshan, and Qianshanyang sites, all in Zhejiang Province, were examined for this study (Figs. 1 and 2). Details are provided in Tables S1 and S2, and Figs. S1 and S2. The stones were preserved in waterlogged contexts and are not charred. The stones assessed in this study, including the sample from Japan, were all preserved under similar anaerobic, wet conditions so that the measurements and ratios are comparable. The archaeological peach stones are discussed in chronological order from oldest to youngest. In total, 202 well-preserved peach stones comprise the sample in this study (Table S2). Stones were measured in three dimensions: length (L), width (W) and suture diameter (Ds; suture diameter is used in order to eliminate the potential confusion between width and thickness). The Ikiriki site peach stone measurements are from Minaki et al. [7]. Specimens from Majiabang were examined but could not be measured because only endocarp fragments are present; however, the exterior surface of the endocarp fragments has grooves and pits similar to those from the other sites so the Majiabang stone identification as *P. persica* is not in question.

## Results

### Peach Botany

**Figure 3:**
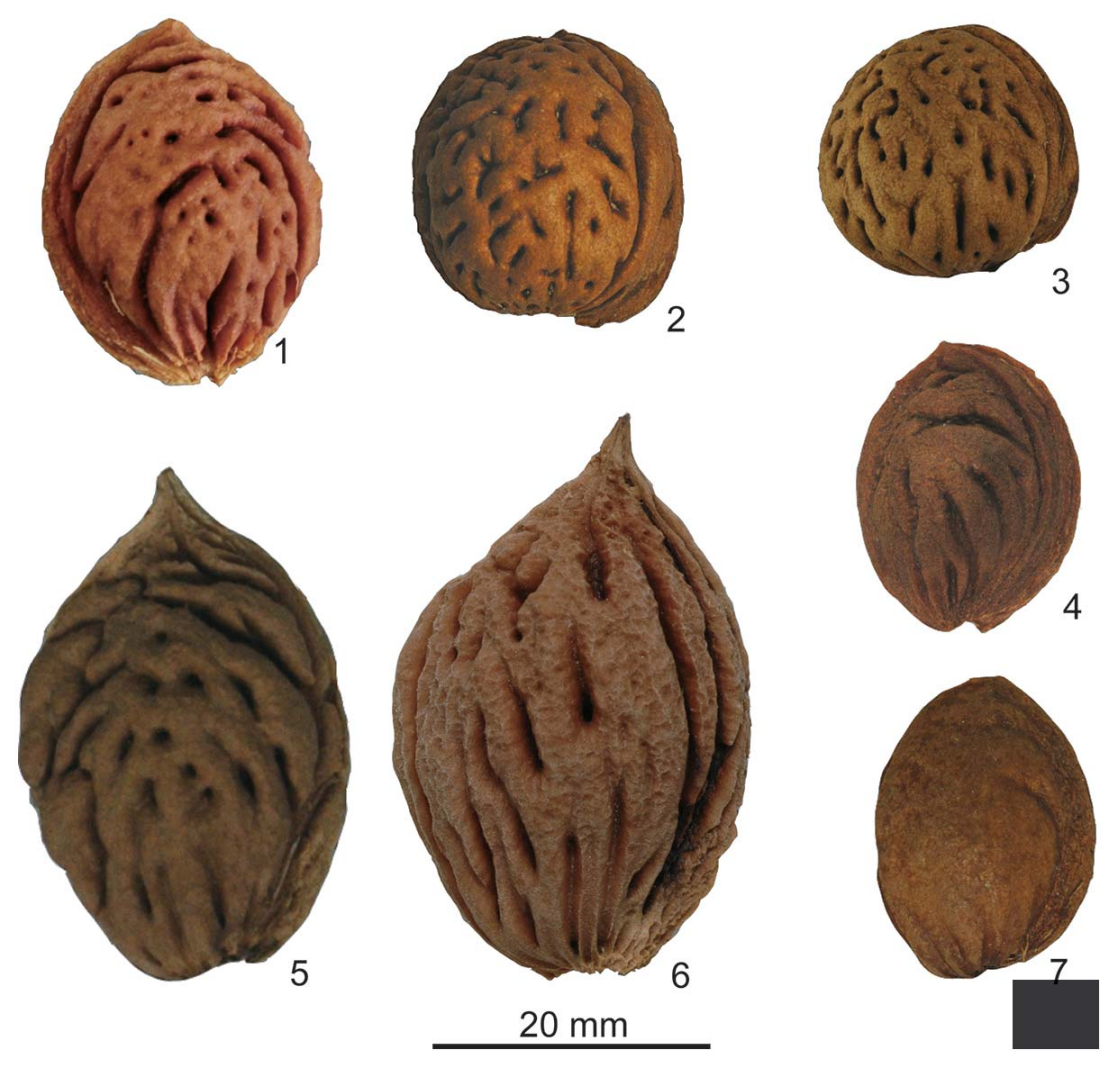
Stones of wild and domesticated peaches. 1. *P. persica* (feral type); 2. *P. davidiana* var. *potaninii*; 3. *P. davidiana* var. *davidiana*; 4. *P. kansuensis*; 5. *P. persica* (domesticated type); 6. *P. ferganensis* (domesticated type from Xinjiang); 7. *P. mira.*

Domesticated peach is a member of the Rosaceae family in the subgenus *Amygdalus* that also includes peach relatives (peach, apricot, nectarine) and almond (*P. dulcis*) relatives [8]. It is a deciduous tree that is known today only in cultivation. It has a wide geographic range, produced in temperate regions such as southern Canada to tropical/subtropical regions (Brazil, Mexico, Yunnan) [8]. In China, three ecological types are recognized: northern, northwestern and southern [9]. The genetic diversity of peach is highest in China with 495 cultivars recognized [10], [11] indicating that peach has a long history there. These data are consistent with a Chinese origin of domesticated peach [12]–[14]. Peach is widely thought to have been domesticated in northwestern [13], [15] or North China [16]. Closely related species are *P. kansuensis* (Tibet and Gansu; Gansu peach), *P. davidiana* (mountain peach), and *P. mira* (Tibetan peach). *P. davidiana* var. *potaninii* (*P. persica* var. *potaninii*) is distinguished from *P. davidiana* var. *davidiana* (*P. persica* var. *davidiana*) by subtle distinctions in the leaves and the shape of the stone (pyrene); the former are more globose while the latter are more ellipsoid to ovate [17] (Fig. 3). *P. ferganensis* is adapted to the dry valleys of central Asia where it is also cultivated [16] and is genetically indistinguishable from cultivated peach, particularly the Shenzhou Mitao cultivar. *P. ferganensis* may be intermediate between wild and cultivated peach or may be a variety of cultivated peach [18]. These taxa are interfertile. The ancestry of cultivated peach, which taxa are feral or truly wild, and how these taxa are related are still open questions. One study proposes that these taxa have an evolutionary relationship, proceeding from *P. mira*, through *P. kansuensis*, *P. davidiana*, *P. ferganensis* and finally *P. persica*; however, the same study cites many outstanding problems and notes that the ancestry of peach is still problematic [15]. Another suggests that the four kindred taxa are all subspecies of *P. persica* [19], [20]. Despite ongoing research on peach origins through morphology, palynology, cytology, biochemistry, and DNA, the specific ancestor of peach is unclear. The *Flora of China* notes that the ancestor is extinct [17].

The peach fruit is a drupe (stone fruit) consisting of a fleshy mesocarp surrounding a stone or pyrene (hard endocarp containing a seed). The thick endocarp promotes long dormancy so peach breeders have developed techniques to break dormancy including scarification of the seed and seed stratification to stimulate germination [21]. The peach seed is bitter and not normally eaten because of the presence of cyanidic glucoside [22]. Peach is a diploid with no recent whole-genome duplication [18]. Flowers are bisexual and self-compatible, out crossing at a rate of about five percent. Thinning of the fruit due to a high fertility rate is required in order for fruit to achieve commercial size [22]. *P. davidiana* is the only one of the wild/feral taxa in China to cross-pollinate with peach. Fruit production begins in the second to third year after germination. Rapid maturation and selfing mean that wild peach had a high biological potential for domestication compared to other trees. Modern peach loses considerable productivity after 10–15 years [22] but early Chinese records report that productivity declined in years 7–8 [10].

Domestication is a complex issue usually referencing a plant's dependence on people for its reproduction (mainly seed dispersal) because it has developed traits (domestication related traits or DRTs) beneficial to people but leave it maladapted to the wild [23], [24]. Such traits comprise the domestication syndrome. Most traits of the domestication syndrome are quantitatively inherited so it is not always clear if and when a plant can be considered domesticated. Even modern domesticated plants continue to undergo selection so the process is never complete. Crossing with interfertile relatives maintains diversity in the crop but can also make it difficult to identify whether a plant is domesticated, wild or intermediate, particularly in the archaeological record. In this paper we use the term “domesticated” relatively loosely to refer to a segregated population of peach trees with traits desirable to people and whose segregation requires human management. These trees can still reproduce on their own.

Cultivated peach stones differ from those of its known wild relatives in being significantly larger and less spheroidal. Other domesticated peach traits include the proportion of mesocarp, proportion of stone size to amount of mesocarp [25]; and selection for varied fruit maturity times to allow for a continuous supply of fruit over a longer period of time [18]. Fruit maturation rate is also a DRT. Wild species are mid- to late-maturing while the crop ripens faster [22]. High nucleotide diversity is found in the region of the genome that controls for fruiting time indicating that breeding has selected for diversity in this region [18]. Other DRTs include slow ripening, flat shape, aborting fruit, and red flesh [26]. Vegetative reproduction facilitates the production of clones with desirable traits. Seed propagation today is normally reserved for the production of rootstock although rootstock is usually produced vegetatively [21].

**Figure 4:**
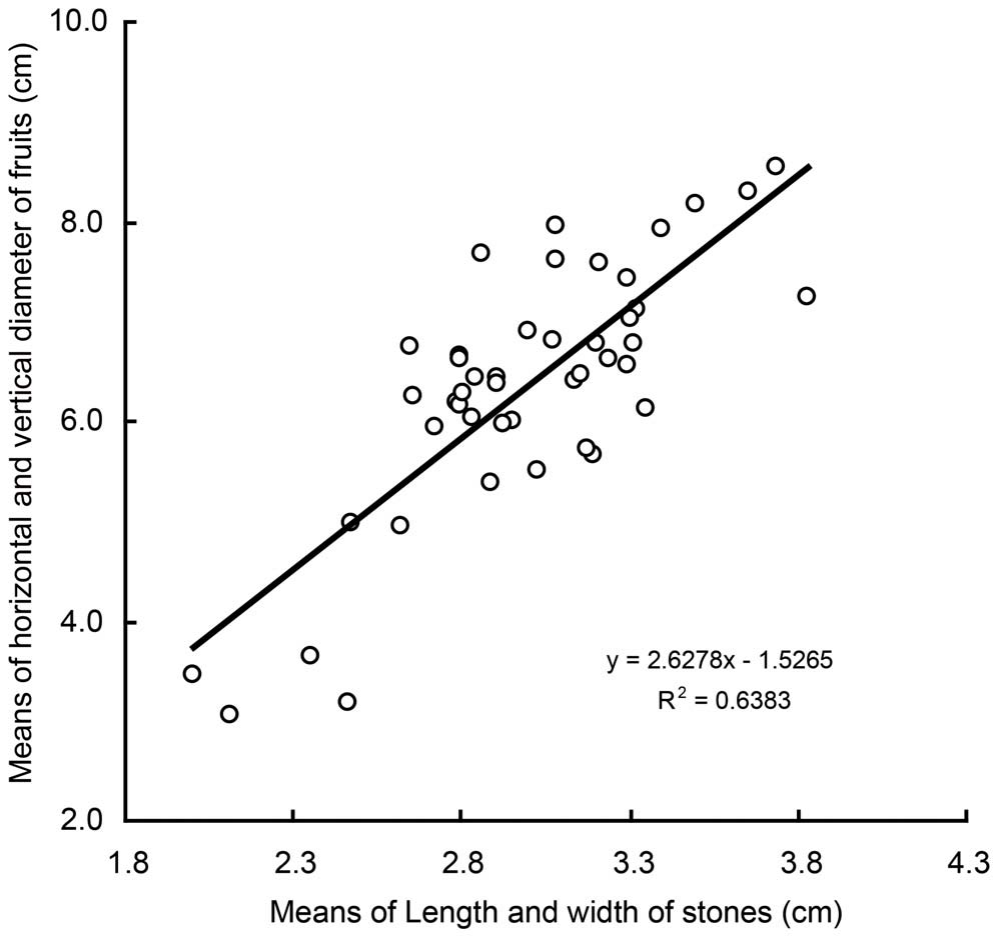
Correlation of stone and fruit sizes in modern peach cultivars. The data are from http://www.ziyuanpu.net.cn/Resource/34/search.html and the feral peaches growing in Zhejiang, China.

Which of these DRTs can be discerned from the archaeological peach stones is problematic. Stone size and shape are the crucial traits to assess in archaeological collections. Stone shape and size are relevant because of their potential relationship to mesocarp thickness. Mesocarp thickness correlates directly with ovalness of the stone in a small sample of peaches [25]. Increased fruit mass positively correlates with degree of domestication in “old” versus “new” [25] cultivars but this may not be the same as the primary domestication process. However the study [25] could not discern a link between stone mass and degree of domestication. Comprehensive data from the Chinese Academy of Agricultural Sciences, however, indicate a positive correlation between peach stone and fruit size [27] (Fig. 4). Stone size is likely a proxy for fruit size while stone shape should also be considered because of its potential relationship to mesocarp thickness. We examine the peach stones to test whether size and shape change over time and when these changes are apparent.

### Archaeological Background

**Figure 5:**
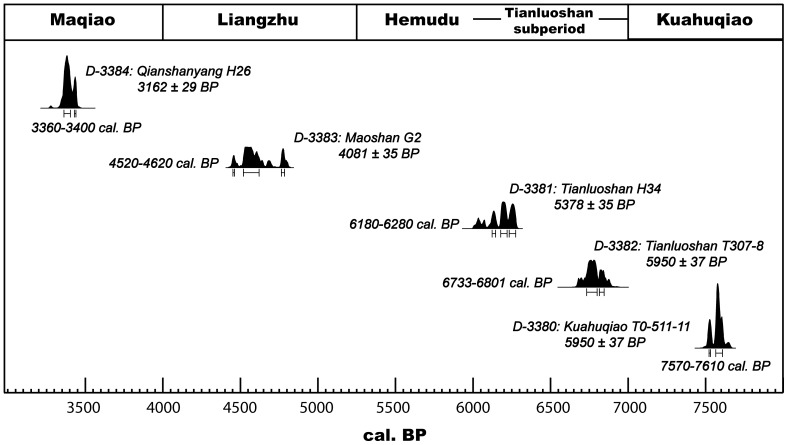
AMS dates on *Prunus* stones. All are *P. persica* except for D-3381 that is *P.* sp., a plum/cherry. Conventional dates were calibrated using Calib 6.0 using the Intcal 09 curve (1, 2).

The oldest peach stones in this study are from Kuahuqiao (ca. 8000–7000 BP) (Figs. 2 and 5). Kuahuqiao is a relatively large and complex occupation with excellent preservation revealing evidence of wood-framed pits for acorn storage, sophisticated pottery technology, stone and bone tools, and the earliest dugout canoe yet found in China. A diverse animal bone assemblage of 34 taxa includes antelope (*Capricornis sumatracnsis*), buffalo (*Bubalus* sp.), pig (*Sus scrofa*), deer (Cervus spp.), dog (*Canis familiaris*), swan (*Cygnus* sp.), wild goose (*Anser* sp.), two species of crane (*Grus *sp.), eagle (*Aquila* sp.), snakehead fish (*Ophiocephalus argus*), carp (*Cyprinus carpio*), alligator (*Alligator* cf. *sinensis*), dolphin (*Delphinidae*), and rhinoceros (*Rhinoceros* sp., although probably *Dicerorhinus sumatrensis*). The dominant plant remains include acorn (*Quercus* sp. and probably mainly *Cyclobalanopsis* sp. and *Lithocarpus* sp.), bramble (*Rubus* sp.), southern sour jujube (*Choerospondias axillares*), peach, plum (*Prunus* sp.), and persimmon (*Diospyros* sp.). Bottle gourd (*Lagenaria siceraria*) is also present. Aquatic plants include water chestnut (*Trapa natans*), fox nut (*Euryale ferox*), and rice, all of which grow in the area today. The rice is an early, cultivated form undergoing selection for domestication related traits (DRTs) such as reduced rachis brittleness [28]–[30]. The peach stones for this research were recovered from strata in vertical excavations. The AMS date on a peach stone from Kuahuqiao confirms its association with this early Neolithic occupation (Fig. 5).

Tianluoshan immediately post-dates Kuahuqiao. Early and late cultural periods date from 7000 to 6500 BP and from 6500 to 5000 BP respectively. A rich variety of artifacts include pottery vessels, stone, wood and bone tools. A large number of upright wooden piles indicate that dwellings and other structures were adapted to a wetland environment. Due to the anaerobic, waterlogged conditions, large quantities of organic remains have been found. Animal remains include buffalo, deer, pig, and fish. Seed and fruit remains included rice, acorns, southern sour jujube, peach, plum, apricot, water chestnut, and foxnut. These provide evidence of a mixed economy of fishing, hunting, gathering and rice cultivation. A large area of rice fields associated with the occupation has been confirmed [31]. Rice took on a greater dietary role over time at Tianluoshan and Hemudu [32]. The peach stones for this research were recovered from stratigraphic layers and pits. Two AMS dates on *Prunus* specimens, one being a cherry or plum, from Tianluoshan bracket the occupation (Fig. 5).

Two Late Neolithic peach stone collections are from the Liangzhu culture Maoshan and Bianjiashan sites. The technology here includes pottery, stone, jade, wood, and other objects. The site consists of a residential area of nearly 30,000 square meters in the south and ca. 55,000 square meters of paddy fields located in the north. The occupation is multi-component consisting of late Majiabang (6300–6000 BP), Songze (5800–5500 BP), Middle Liangzhu (4900–4600 BP), late Liangzhu (4600–4300 BP, and Guangfulin (4300–4000 BP) cultures. The AMS date on a peach stone from Maoshan is consistent with the Middle Liangzhu period (Fig. 5). Social complexity is well developed [33], evidenced by public architecture and elaborate burials. The economy was substantially agricultural. The peach stones for this research were recovered from a small river (in unit G2). Other seeds recovered from the same context include rice, bottle gourd, melon, water chestnut, foxnut, plum, apricot, southern sour jujube, and Chinaberry. Bianjiashan is a late Liangzhu culture site (ca. 4500–4400 BP) (Fig. 5). The site consists of tombs, houses, ditches, pits, wood piles, and building components as well as a rich assemblage of pottery, stone, jade, wood, bone, lacquer ware, and bamboo weavings/matting. Organic remains are well preserved. Animal remains include pig, deer, and buffalo. The abundant plant remains include rice, water chestnut, foxnut, peach, apricot, plum, bottle gourd, melon, southern sour jujube, and acorn.

The latest peach stone population in this study is from the Qianshanyang site. The site is late Neolithic and Bronze Age spanning the Qianshanyang (4200–3900 BP), and Maqiao (3900–3500 BP) cultures [34]. The latter culture is contemporary with Middle and Late Shang. Large quantities of pottery, stone, bone, wood, and textiles/fabrics plaited with natural silk have been recovered. The settlement remains consist of houses with postholes and wall foundations and numerous pits. The plant remains include rice, bottle gourd, melon, water chestnut, foxnut, plum, apricot, southern sour jujube, and Chinaberry. The peach stones for this research are from the Maqiao period pits. The AMS date on a peach stone from Qianshanyang confirms that the stones are associated with the Maqiao occupation of the site (Fig. 5).

The Ikiriki site, Kyushu, Japan is stratified with Early, Late and Final Jomon occupations as well as a Yayoi component. It is a well-preserved wet site on the coast about 800 km from the Yangzi River mouth. Forty-one plant taxa and a dugout canoe are reported [27]. Ten peach stones (eight with relatively complete measurements) are from the Early Jomon levels (VII and VIII) while eight are from later Jomon contexts [27]. Jomon subsistence is complex and varied, including hunting, fishing, gathering, resource management, limited crop production depending on the region and period and includes a wide range of anthropogenic habitats [35], [36].

### Archaeological Peach Stone Analysis

Stones of cultivated peach have deep furrows and pits and are compressed or ovate, unlike those of wild taxa in China (Fig. 3). Our examination of the stones of the closely related taxa confirms published descriptions [21], [37]. Modern *P. persica* stones are distinct from both types of *P. davidiana* in having small pits and curved furrows, an acute apex (an extension of the hilum), a round base, and an elliptical hilum running 180 degrees along the suture. The archaeological specimens are more variable than modern peach stones but they all have furrows and pits. The earliest stones, from Kuahuqiao, have the greatest variation of furrow and pit forms and have a subtle, acute apex (Fig. 2). *P. ferganensis* stones are most similar to *P. persica* stones on the basis of size and shape but pits are absent (Fig. 3). Other significant characteristics of peach stones include their dimensions (length, width, suture diameter/thickness). The archaeological specimens are most similar to the reference specimens of *P. persica*.

**Figure 6:**
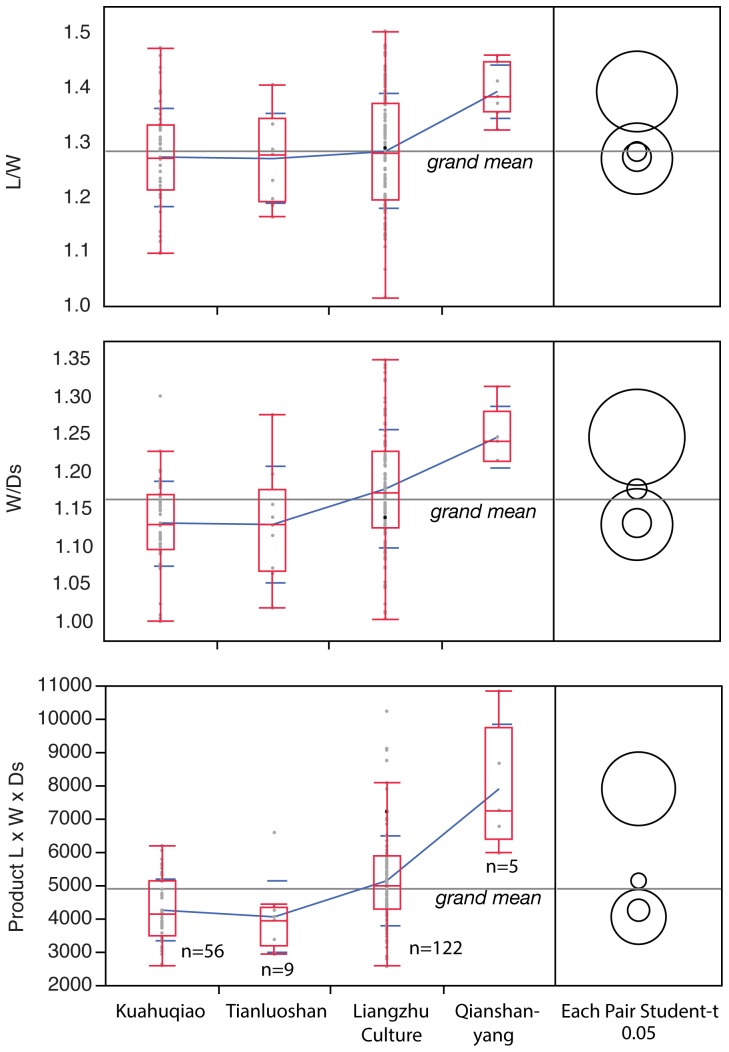
Box plots of peach stone size (L×W×Ds) and ratios of L, W, and Ds. The plots illustrate an overall trend to the ratios diverging from 1∶1 through time and of approximate stone volume increasing through time. The top, bottom and line through the middle of the box correspond to the 75th percentile (top quartile), 25th percentile (bottom quartile) and 50th percentile (median) respectively. The whiskers on the bottom extend from the 10th percentile (bottom decile) and top 90th percentile (top decile). Means are joined. The circles are a graphic representation of Student's t-test results. The concentric circles indicate that the means are not significantly different. The Liangzhu culture plot is a combination of the Maoshan (n = 99) and Bianjiashan (n = 23) measurements.

The approximate mean volume (or proxy for volume) of the stones represented by the product of the three measured dimensions (L×W×Ds) nearly doubles between ca. 8000 and 3700 BP (Fig. 6). The mean proxy volumes of the two oldest populations (Kuahuqiao and Tianluoshan) are not significantly different but that of the Maoshan population is significantly larger. Most of the Qianshanyang stones are larger than the stones from all earlier periods.

The shapes of the peach stones are described by three ratios of L, W, and Ds (Fig. 6 and Fig. S2). Ratios of 1∶1 indicate a spheroidal shape while deviations from this ratio indicate the extent to which the stones are compressed and/or ovoid. In fact, the mean ratios indicate that the two early samples are more spheroidal on the whole than the later samples with the Qianshanyang assemblage having no spheroidal specimens. The other stone assemblages grade from spheroidal through flattened/ovate. The Qianshanyang sample is the only sample whose means are significantly different from the others. The W/Ds ratio for the Maoshan stone shape has deviated significantly from the shape of the large Kuahuqiao sample. Thus the shape trends to ovate/compressed through time.

**Figure 7:**
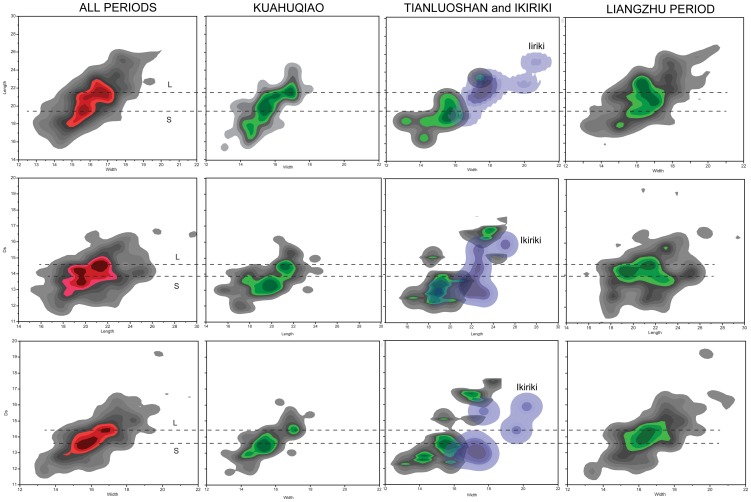
Contour map of scatterplot of Ikiriki, Kuahuqiao, Tianluoshan and Liangzhu Culture (Maoshan and Bianjiashan sites combined) peach stone measurements. The colors emphasize the highest three contours (the peaks) of each scatterplot. The dashed lines indicate the peaks for the smaller (S) and larger (L) stones. 1. Reimer PJ, *et al.* (2011) *IntCal09 and Marine09 Radiocarbon Age Calibration Curves, 0–50,000 Years cal BP*. 2. Stuiver M & Reimer PJ (1993) *Extended 14C data base and revised CALIB 3.0 (super 14) C age calibration program*.

Visual inspection suggested that the Kuahuqiao stone population is composed of two types on the basis of shape and size: a smaller spheroidal type and a larger, compressed/ovate type. The measurements seem to confirm that some of the early specimens are compressed/ovate, particularly those with high L/W ratios (Fig. 6). A contour density plot of the scatterplots teases out some details of the bimodal distribution (Fig. 7). Two high density regions of the scatterplots are clear for all measurements although L vs Ds has a minor third region suggesting a trimodal distribution for those measurements (Fig. 7). Fig. 7 also breaks out the scatterplots by site. The latest assemblages have a single, larger mode. The Tianluoshan sample, despite having only nine measured specimens, also separates into two modes similar to the sample from Kuahuqiao but in this case the two types do not overlap. The Maoshan and Bianjiashan stones are the compressed, ovate type with an acute apex. Their contour density plots of the scatterplots show single density peaks of the measurements that also have considerable variation. Finally, the five Qianshanyang stones are unimodal (not illustrated), similar to those from Maoshan. Most of the Ikiriki specimens fit within the large group (Fig. 7).

## Discussion

Tree fruit have not played a significant role in our understanding of human-plant interaction that led to food production because their domestication is usually considered to be much later than the domestication of herbaceous, annual plants such as grains and legumes. Examples of tree fruit include olive, date and fig that were important resources in the eastern Mediterranean by ca. 6000 BP [3]. Olives were collected as early as 19,000 BP at Ohalo II along with almond, pistachio, and grape [38] so considerable time passed until they were domesticated. A strong case has been made for fig domestication in Israel 12,000–11,000 BP by vegetative reproduction before the domestication of large-grain grasses in SW Asia [4]. In Japan, chestnut and lacquer tree management (grown not for fruit but for resin and wood) has been documented [36]. The management techniques are not clear but controlled burning for acorn production has a long history in North America, particularly California [39], [40]. However, biological traits of many tree taxa, particularly nut bearing trees, olive, and date, militate against their domestication; their exploitation is not viewed as a pathway to agriculture [41], but as a result of agricultural systems and related plant breeding knowledge. For example, trees have a long juvenile stage thereby discouraging reproduction from seed; nut trees also do not produce significant masts every year. Furthermore most cross-pollinate, making segregation of preferred varieties difficult [3], [41]. Tree domestication is generally thought not to have emerged until people started cloning trees through vegetative reproduction [3] but cloning is not particularly difficult.

Domesticated peach self-pollinates; only *P. davidiana* among the members of the *Amygdalus* subgenus in China cross-pollinates; at least one cultivar, J.H. Hale, cross-pollinates due to male sterility [21]. Preferred traits could be selected without vegetative reproduction if the wild ancestor of peach also self-pollinated, then preferred traits could be selected without vegetative reproduction. If selfing developed after domestication, as has been suggested for olive [3], then vegetative propagation would have played a bigger role in peach breeding at the outset; it may have anyway. Peach matures quickly, producing fruit starting in the second or third year, so selection for desirable traits based on this trait alone has a higher probability for a quick payoff compared to olive and date. Larger fruited populations, for example, could be readily segregated and selection further enhanced by rootstock production and grafting. Furthermore, *Prunus* spp. are generally fire tolerant [16] so initial selection by local burning may have encouraged stands of peach. Burning was already an ecological management tool at Kuahuqiao [42], [43] and we have no reason to believe that controlled burning was unique to that community in eastern China.

All indications are that relatively sedentary food producing, hunting, fishing, gathering societies were well established in China by 8000–7500 BP. Settlements with some degree of sedentism appear in the region between 10,000 and 8000 BP (Shangshan culture). Some plants such as millet were domesticated by this time and at least one wild ancestor of a millet appears to have been utilized during the late Upper Palaeolithic [44], [45]. Other plants such as soybean were being harvested but all indications are that their domestication was a prolonged and complex process not limited to China; soybean appears not to have been domesticated as early as millet [46]. Rice production was in its early stages at Kuahuqiao and a range of plants that would eventually be important crops in China was being utilized. The first archaeological evidence for peach is also from this context.

All plant taxa recovered from Kuahuqiao grow in the region today so we have no reason to believe that peach was an exception. The lower Yangzi basin climate is maritime subtropical today with cool, dry winters and warm, wet summers. Kuahuqiao was occupied at the beginning of the Hypsithermal period when average annual temperatures rose to about 2.5 degrees Celsius above late 1990s levels [47] and it was somewhat wetter than in the preceding Early Holocene. Forest cover represented in pollen Zone 2 in the Lower Yangzi included more broadleaf evergreen (subtropical) taxa but deciduous taxa were still common [43], [48]. Rhinoceros and elephant (*Elephas maximus*) expanded their ranges northward into Zhejiang province. Rhinoceros bones have been identified at sites such as Kuahuqiao [49] and elephant is reported among the remains at Hemudu [50]. After 3800 BP subtropical taxa decrease significantly in the region [48]. Furthermore, the common occurrence of peach remains at sites in the Lower Yangzi is clear evidence that it continued to flourish there.

The tree fruit taxa being collected at Ohalo II ca. 19,000 cal. BP. and the fig that was probably domesticated 7000 years later indicate that tree fruit collection was not an exclusively Holocene human activity. [4]. We cannot assume the 8000–7000 BP peach stones in China are evidence of their first use. Diverse groups before this likely collected peach and were selecting for preferred traits by 8000–7500 BP. Three of the earliest peach stone assemblages are outside the lower Yangzi, one in the Huanghe valley (Egoubeigang) and two in the Middle Yangzi Basin (Hujiawuchang and Bashidang) so that peach was being utilized over a broad geographic area by 8000–7000 BP.

The surface sculpting and general morphology of the archaeological specimens examined here are consistent with the stones being *P. persica. P. davidiana* stones are more spheroidal than the stones in our analysis. Stones from the earliest assemblages at Kuahuqiao and Tianluoshan further differ from those of *P. davidiana* in having a range of short, shallow furrows to deep and long furrows and pits combined with a slightly acute apex (Figs. 4 and 5). The evidence so far indicates that *P. davidiana* is probably not the ancestor of *P. persica*. If *P. davidiana* is the ancestor of *P. persica*, the differentiation took place well before 8000 BP.

The earliest assemblages appear to be bimodal and we hypothesize that some fruit was being selected for greater mass of mesocarp and possibly fruiting time so that selection for preferred traits was under way. *P. davidiana* fruit is bitter and rather unpalatable making it far less appealing than the sweet fruit of *P. persica*. The oldest small specimens in this study are consistent with a “truly wild peach (that) no longer exists” [16]. The apparent bimodality of the earliest peach stone assemblages suggests two hypotheses: 1) two types of peach, a wild form with comparatively small amounts of mesocarp and a cultivated form with a comparatively large amount of mesocarp, comprised the populations at Kuahuqiao and Tianluoshan or 2) the wild peach population consisted of a wide range of peach forms from which the domesticated types evolved by Liangzhu culture times. Given the bimodal nature of the peach stones that becomes more prominent by Tianluoshan times the second hypothesis seems less likely.

In the subsequent millennium at Tianluoshan, the measured sample numbers only nine so the sample is too small to effectively document the variation of peach stone morphology at this time. The small sample appears to split into two groups similar to the sample from Kuahuqiao. The set of peach stone measurements from the Early Jomon levels at the Ikiriki site in Japan indicate that peach between 7000 and 6000 BP was, indeed, being differentiated from the wild form. Their mean approximate volume is similar to that of the Maoshan sample. The earliest peach stones are associated with radiocarbon dates from level VIII that range from 6700–6400 cal. BP [27]. The W/Ds ration is 1.3, indicating significant compression, more than the Bronze Age Qianshanyang sample exhibit. The source of these peach stones in China cannot be determined yet, but if the dating is correct then apparently peach was domesticated by 6400 BP. Peach was introduced to Japan, evidenced at the coastal Ikiriki site, a distance of about 800 km from the Yangzi river mouth. A few more stones, somewhat larger and more compressed than the Early Jomon specimens, have been recovered from later Jomon deposits at the site indicating some continuity in their use. The only other Jomon period peach stones are from Late Jomon deposits at Nabatake, also in Kyushu [51]. The early appearance of apparently domesticated peach in Japan where wild relatives of peach are not known indicates that peach was of special significance and so, by inference, was cultivated in China by at least 6700–6400 BP. It was in widespread use in China by 4000 BP and appears in India by ca. 3700 BP [52]. The Liangzhu culture (5300 BP-4300 BP) and Maqiao culture (4000 BP-3700 BP) peach stones are more characteristic of the domesticated peach than are their earlier counterparts.

The domestication process conservatively took at least three millennia considering only the Yangzi valley peach record. However, the process may well have been considerably faster considering the Early Jomon (Japan) data that indicate a founding population of a distinct type of peach from an as yet unidentified locale in China. Our sample from the same period in China is as yet too small to provide meaningful insight on this question.

The archaeological record is at odds with the current wisdom regarding its domestication, particularly the notion that it was domesticated in northwestern China. The archaeological database for peach has long been recognized as confirming the view that the peach had a long association with people in China [10]. The first written reference to peach is found in China's earliest agricultural almanac, Xiaxiaozheng, which refers to the Xia Dynasty (ca. 4100–3600 BP) [47] while the *Shijing* (*Book of Odes*, a compilation of poetry spanning the period ca. 3000 BP–2500 BP) has the earliest botanical description of peach [53]. Peach has been an important aspect of traditional culture in China, and was considered a symbol of immortality in Daoist mythology [54] while the fruits are important gifts [55], [56]. Twelve of the Chinese sites from which peach stones have been recovered are in the lower Yangzi River valley. Most of the other specimens are from the middle and upper reaches of the Yangzi valley or from southern and southwestern China. Peach in the form of charred stones is reported from only three sites in North China where the only wet site reported is Jiahu. Peach remains have not been reported from Jiahu. Peach stones preserve well after charring; flotation to recover charred plant remains is relatively common in the north. If peach were economically significant there we would expect better representation in the archaeological record, including at Jiahu. Although preservation bias may be a factor in the few reports of peach from North China, given the extensive sampling there and the research at Jiahu, peach was likely not a significant resource there during the Early Neolithic.

Not until about 4000 BP does the distribution of archaeological peach stones extend west of the Hujiawuchang site or north of Egoubeigang although peach had reached Japan by 6500 BP. The data reported here provide more specific documentation of a long developmental sequence in eastern China that resulted in the plant's domestication. The commonly cited northwestern China region can be ruled out as the region where peach was domesticated. Although North China cannot be categorically ruled out, the most feasible working hypothesis is that peach was domesticated in the Yangzi valley. Finally, tree fruit management and domestication undoubtedly played a significant role in the early phases of agricultural development in China as well as other parts of the world.

## Supporting Information

Figure S1 **Geographic distribution of archaeological peach remains.** The site number key is in Table 1. •, Neolithic; ○, Historic.
(TIF)

Figure S2 **Box plots of the L/Ds ratio illustrating an overall trend diverging from 1∶1 through time.**
(TIF)

Table S1 **Archaeological sites from which peach stones are reported.**
(DOCX)

Table S2 **Peach stone measurements.**
(DOCX)
