# Supplementary figures and images for: Archaeological Evidence for Peach (Prunus persica) Cultivation and Domestication in China
Source: PLoS One. 2014 Sep 5;9(9):e106595. doi: 10.1371/journal.pone.0106595 (PMC4156326; doi:10.1371/journal.pone.0106595)

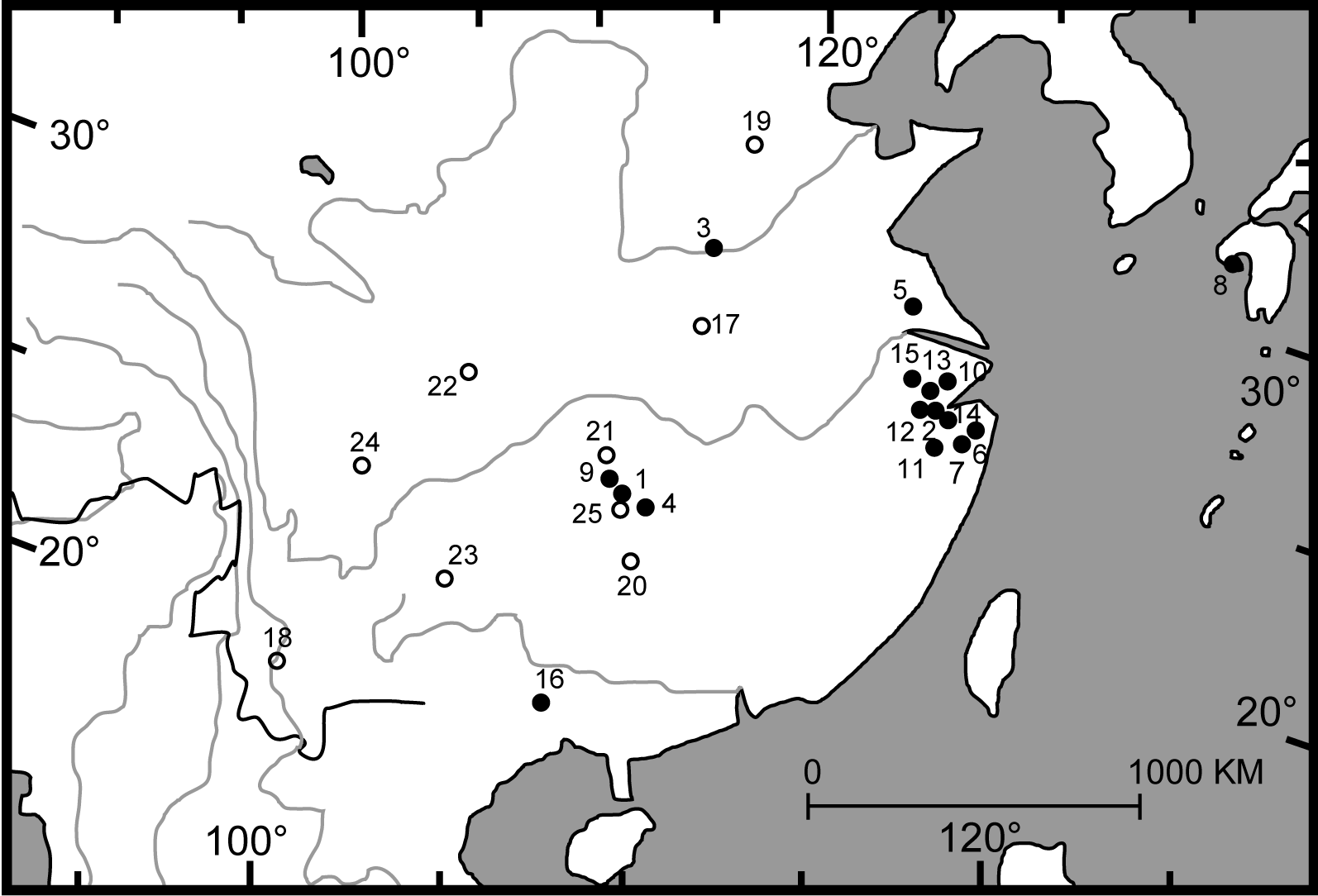

Supplement: Figure S1 — Geographic distribution of archaeological peach remains. The site number key is in Table 1. •, Neolithic; ○, Historic. (TIF) [file pone.0106595.s001.tif]

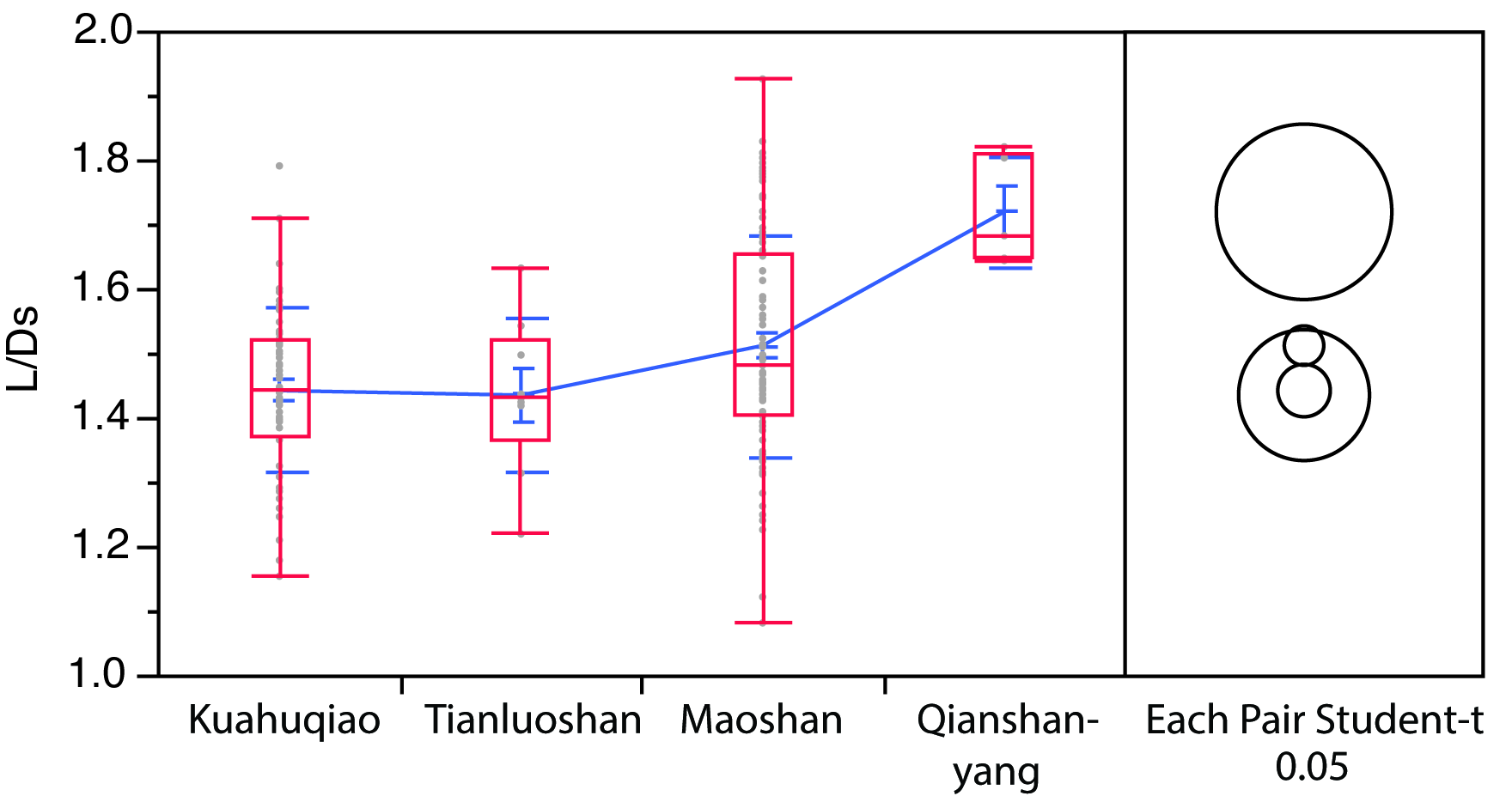

Supplement: Figure S2 — Box plots of the L/Ds ratio illustrating an overall trend diverging from 1∶1 through time. (TIF) [file pone.0106595.s002.tif]
