# Supplementary material for: Archaeological Evidence for Peach (Prunus persica) Cultivation and Domestication in China
Source: PLoS One. 2014 Sep 5;9(9):e106595. doi: 10.1371/journal.pone.0106595 (PMC4156326; doi:10.1371/journal.pone.0106595)
Supplement: Table S1 — Archaeological sites from which peach stones are reported. (DOCX) [file pone.0106595.s003.docx]

| **Number** | **Sites** | **cal. BP** | **Period/Culture** | **Source** |
| --- | --- | --- | --- | --- |
| 1 | Bashidang | 8500-7600 | Early Neolithic, Pengtoushan | [1] |
| 2 | Kuahuqiao* | 8000-7500 | Early Neolithic, Kuahuqiao | [2] |
| 3 | Egoubeigang | 8000-7000 | Early Neolithic | [3] |
| 4 | Hujiawuchang | 7900-7300 | Early Neolithic | [4] |
| 5 | Qingdun | 7400-6400 | Early Neolithic | [5] |
| 6 | Hemudu | 7000-6500 | Middle Neolithic, Hemudu | [6] |
| 7 | Tianluoshan* | 7000-6500 | Middle Neolithic, Hemudu | [7] |
| 8 | Ikiriki, Japan* | 6700-6400 | Early Jomon, levels VII-VIII | [8] |
| 9 | Chengtoushan | 6400-6100 | Early Daxi | [9] |
| 10 | Majiabang | 6000 | Middle Neolithic, Majiabang | [10] |
| 11 | Shuitianfan | 4500 | Late Neolithic, Liangzhu | [11] |
| 12 | Bianjiashan* | 4300 | Late Neolithic, Liangzhu | [12] |
| 13 | Maoshan* | 4300 | Late Neolithic, Liangzhu | [13] |
| 14 | Jianshanwan | 4300 | Late Neolithic, Liangzhu | [14] |
| 15 | Qianshanyang* | 4300-3500 | Late Neolithic, Liangzhu | [15,16] |
|  |  |  | Bronze Age, Maqiao |  |
| 16 | Duliao | 4000 | Late Neolithic, Liangzhu | [17] |
| 17 | Yangzhuang | 4000-3600 | Xia and Shang | [18] |
| 18 | Baiyangcun | 3700-3100 | Shang | [19] |
| 19 | Taixi | 3700-3100 | Shang | [20] |
| 20 | Tombs | 770-475 BC** | Eastern Zhou | [21] |
| 21 | Tombs | 475-221 BC | Warring States | [22] |
| 22 | Tombs | 475-221 BC | Warring States | [23] |
| 23 | Tombs | 475-221 BC | Warring States | [24] |
| 24 | Tombs | 475-221 BC | Warring States | [25] |
| 25 | Tombs | 475-221 BC | Warring States | [26] |

* Measurements included in this study

** Historic dates represented by BC

1. Yi JS, Gu HB, Tan YH (2006) Pengtoushan and Bashidang (in Chinese). Beijing: Science Press.

2. Jaing LP, Zheng YF, Fang XM (2004) Kuahuqiao; Jiang L, editor. Beijing: Cultural relics Publishing House.

3. Yang ZQ (1981) A Neolithic site at Egou, Henan (Chinese). Papers of Chinese Archaeology 1.

4. Wang WJ, Zhang CL (1993) The Neolithic site at Hujiawuchang, Linli County, Hunan (Chinese). Acta Archaeologica Sinica 2.

5. Ji QZ (1983) A Neolithic site at Qingdun, in Haian County, Jiangsu Province (Chinese). Acta Archaeologica Sinica 2: 147-190.

6. Liu J, Yao ZY, Mei FG (2003. ) Hemudu (Chinese). Beijing: Cultural Relics Publishing House.

7. Sun GP, Huang WJ, Zheng YF ( 2007) A brief report of the excavation on a Neolithic site at Tianluoshan Hill in Yuyao, Zhejiang (Chinese). Cultural Relics 11.

8. Minaki M, Hohjo S, Kokawa S, Kosugi S, Susuki M (1986) Plant remains and ancient environment. In: iinkai T-cK, editor. Ikiriki Iseki: Tarami-cho Kyoiku Iinkai. pp. 44-53.

9. Yasuda Y, Fujiki T, Nasu H, Kato M, Morita Y, et al. (2004) Environmental archaeology at the Chengtoushan site, Hunan Province, China, and implications for environmental change and the rise and fall of the Yangtze River civilization. Quaternary International 123-125: 149-158.

10. Yao ZY, Mei FG (1961) Excavation of the Neolithic site at Ma Chia Ping, Chiahsing Country, Chekiang. Archaeology 7: 345-351.

11. Mei FG (1960) The excavation of Shuitianfan site in Hangzhou (Chinese). Acta Archaeologica Sinica 2: 93-106.

12. Zhao Y (2003) The wooden pier and other traces of the Liangzhu period found at Bianjiashan site. China Cultural Relics News September 27.

13. Ding P, Zheng YF, Chen XG (2010) Maoshan site at Linping, Zhejiang (Chinese). China Cultural Relics News April 6.

14. Jiang LP, Sun GP, JM Zheng (2010) Loujiaqiao, Kuotangshanbei, Jianshanwan. -Archaeological Report of Puyang River Valley (in Chinese). Beijing: Cultural Relics Publishing House.

15. Chang K-C, Pingfang X (2005) The Formation of Chinese Civilization: An Archaeological Perspective. New Haven: Yale University Press. xiv, 363 p. p.

16. Ding P (2010) The Third Excavation of the Qianshanyang Site in Huzhou, Zhejiang. Cultural Relics 7: 4-26.

17. Yu FZ, Fang YZ (1982) Excavation of a Neolithic site at Duliao, Xinzhou, Guizhou (Chinese). Archaeology 1.

18. Song YQ, Li YD, Han JY (1995 ) A preliminary report on the excavation of the site at Yangzhuang, Zhumadian City, Henan (Chinese). Archaeology (Kaogu) 10: 873-882.

19. Kan Y (1981) The Baiyangcun site at Bingchuan County, Yunnan Province (Chinese). Acta Archaeologica Sinica 3: 349-368.

20. Li JM, Hua XR, Wen QM (1974) The Shang Dynasty site at Taihis-tsun, Kau-cheng County, Hopei Province (Chinese). Cultural Relics 8: 42-29.

21. Xu CQ, Yu JA, Hu S (2009) Excavation of Lizhou ’ao Eastern Zhou tombs in Jiangxi (Chinese). Cultural Relics 2.

22. Xiong YY, Ding TH (1983) Chu State tombs in the Suburbs of Echeng (Chinese). Acta Archaeologica Sinica 2.

23. Feng HJ, Yang YR, Wang JY (1958) Ancient burial with canoe coffins in Sichuan Province (Chinese). Acta Archaeologica Sinica 2: 77-95.

24. Song SK, Tang WY, Xiong SF (1986) Excavations at Kele Township, Hezhang County, Guezhou Province (Chinese). Acta Archaeologica Sinica 2 199-251.

25. Yuan J (2006) Funeral objects over 100 from the canoe coffin of the Warring States period at Pujiang County, Sichuan Province (Chinese). wwwxinhuanetcom, December 7.

26. Gao ZX, Chai HB (1995) Tombs of the Warring States period at Shiban Village, Cili County, Hunan (in Chinese). Acta Archaeologica Sinica 2: 173-207.
