# Supplementary material for: Archaeological Evidence for Peach (Prunus persica) Cultivation and Domestication in China
Source: PLoS One. 2014 Sep 5;9(9):e106595. doi: 10.1371/journal.pone.0106595 (PMC4156326; doi:10.1371/journal.pone.0106595)
Supplement: Table S2 — Peach stone measurements. (DOCX) [file pone.0106595.s004.docx]

| **Site Name** | **Length (mm)** | **Width (mm)** | **Suture Diameter (mm)** |
| --- | --- | --- | --- |
| Kuahuqiao | 20.00 | 15.74 | 13.21 |
| Kuahuqiao | 20.74 | 17.20 | 14.55 |
| Kuahuqiao | 21.88 | 18.00 | 15.40 |
| Kuahuqiao | 19.47 | 15.78 | 13.63 |
| Kuahuqiao | 17.01 | 15.07 | 13.49 |
| Kuahuqiao | 19.41 | 15.43 | 13.09 |
| Kuahuqiao | 20.45 | 15.22 | 13.59 |
| Kuahuqiao | 22.51 | 15.26 | 13.72 |
| Kuahuqiao | 21.13 | 16.18 | 14.33 |
| Kuahuqiao | 24.01 | 17.24 | 14.99 |
| Kuahuqiao | 21.48 | 16.91 | 15.23 |
| Kuahuqiao | 21.40 | 16.46 | 14.77 |
| Kuahuqiao | 19.91 | 13.63 | 12.70 |
| Kuahuqiao | 17.98 | 14.56 | 11.85 |
| Kuahuqiao | 18.33 | 15.10 | 13.82 |
| Kuahuqiao | 23.32 | 16.94 | 13.01 |
| Kuahuqiao | 16.59 | 14.60 | 12.89 |
| Kuahuqiao | 19.09 | 14.70 | 12.77 |
| Kuahuqiao | 21.42 | 16.88 | 15.67 |
| Kuahuqiao | 21.19 | 16.01 | 13.67 |
| Kuahuqiao | 18.60 | 15.06 | 13.33 |
| Kuahuqiao | 17.76 | 14.80 | 13.56 |
| Kuahuqiao | 19.58 | 15.55 | 13.59 |
| Kuahuqiao | 17.49 | 14.50 | 13.53 |
| Kuahuqiao | 17.95 | 16.33 | 14.07 |
| Kuahuqiao | 21.50 | 15.40 | 14.00 |
| Kuahuqiao | 18.58 | 15.46 | 14.39 |
| Kuahuqiao | 17.53 | 15.66 | 14.05 |
| Kuahuqiao | 15.34 | 13.07 | 13.00 |
| Kuahuqiao | 24.26 | 16.86 | 14.18 |
| Kuahuqiao | 18.64 | 14.04 | 12.70 |
| Kuahuqiao | 16.78 | 14.75 | 12.28 |
| Kuahuqiao | 20.62 | 16.21 | 16.19 |
| Kuahuqiao | 20.98 | 15.50 | 13.14 |
| Kuahuqiao | 20.04 | 14.41 | 13.15 |
| Kuahuqiao | 20.88 | 16.99 | 14.59 |
| Kuahuqiao | 22.22 | 16.65 | 15.19 |
| Kuahuqiao | 17.24 | 14.11 | 12.08 |
| Kuahuqiao | 18.39 | 15.53 | 13.27 |
| Kuahuqiao | 19.78 | 15.24 | 14.14 |
| Kuahuqiao | 18.38 | 14.28 | 12.94 |
| Kuahuqiao | 15.76 | 14.36 | 13.01 |
| Kuahuqiao | 17.84 | 14.00 | 12.72 |
| Kuahuqiao | 20.13 | 14.08 | 13.94 |
| Kuahuqiao | 19.87 | 14.98 | 12.97 |
| Kuahuqiao | 16.89 | 13.30 | 11.79 |
| Kuahuqiao | 21.05 | 15.77 | 14.39 |
| Kuahuqiao | 21.67 | 16.80 | 14.62 |
| Kuahuqiao | 21.59 | 16.08 | 13.76 |
| Kuahuqiao | 16.30 | 14.46 | 14.11 |
| Kuahuqiao | 21.60 | 17.31 | 14.39 |
| Kuahuqiao | 22.35 | 17.05 | 14.18 |
| Kuahuqiao | 19.81 | 15.18 | 13.44 |
| Kuahuqiao | 22.76 | 17.11 | 14.44 |
| Kuahuqiao | 20.82 | 15.29 | 13.15 |
| Kuahuqiao | 19.81 | 16.05 | 13.34 |
| Tianluoshan | 23.28 | 17.44 | 16.26 |
| Tianluoshan | 21.00 | 15.51 | 13.60 |
| Tianluoshan | 20.21 | 15.80 | 12.37 |
| Tianluoshan | 19.00 | 16.03 | 13.38 |
| Tianluoshan | 19.10 | 15.51 | 13.40 |
| Tianluoshan | 18.45 | 13.11 | 12.31 |
| Tianluoshan | 18.45 | 14.32 | 12.83 |
| Tianluoshan | 18.41 | 15.37 | 15.08 |
| Tianluoshan | 16.52 | 14.19 | 12.56 |
| Maoshan | 22.86 | 16.09 | 13.29 |
| Maoshan | 19.98 | 15.62 | 13.22 |
| Maoshan | 22.07 | 18.30 | 15.29 |
| Maoshan | 21.42 | 16.56 | 14.05 |
| Maoshan | 23.52 | 17.72 | 15.59 |
| Maoshan | 24.84 | 17.41 | 14.04 |
| Maoshan | 21.62 | 18.30 | 15.14 |
| Maoshan | 24.78 | 17.88 | 13.92 |
| Maoshan | 22.09 | 17.27 | 14.63 |
| Maoshan | 24.82 | 17.97 | 14.63 |
| Maoshan | 19.37 | 16.43 | 14.52 |
| Maoshan | 24.55 | 20.88 | 15.79 |
| Maoshan | 20.18 | 16.38 | 13.75 |
| Maoshan | 23.95 | 18.55 | 16.27 |
| Maoshan | 17.75 | 15.51 | 14.46 |
| Maoshan | 23.33 | 16.71 | 13.81 |
| Maoshan | 21.44 | 17.58 | 15.27 |
| Maoshan | 23.54 | 17.82 | 13.48 |
| Maoshan | 22.48 | 15.25 | 13.93 |
| Maoshan | 21.72 | 14.81 | 13.11 |
| Maoshan | 19.55 | 15.85 | 13.43 |
| Maoshan | 21.58 | 16.28 | 14.69 |
| Maoshan | 18.91 | 15.50 | 13.48 |
| Maoshan | 22.90 | 16.32 | 13.62 |
| Maoshan | 19.74 | 16.69 | 12.42 |
| Maoshan | 22.40 | 16.55 | 12.41 |
| Maoshan | 17.98 | 14.97 | 13.69 |
| Maoshan | 17.90 | 15.10 | 13.43 |
| Maoshan | 22.80 | 15.85 | 11.83 |
| Maoshan | 19.84 | 16.09 | 13.62 |
| Maoshan | 18.44 | 16.16 | 14.36 |
| Maoshan | 19.24 | 16.69 | 14.31 |
| Maoshan | 18.30 | 15.19 | 12.80 |
| Maoshan | 22.02 | 17.92 | 14.00 |
| Maoshan | 17.79 | 13.27 | 12.24 |
| Maoshan | 19.93 | 16.66 | 14.29 |
| Maoshan | 18.69 | 14.36 | 12.53 |
| Maoshan | 20.08 | 17.13 | 15.17 |
| Maoshan | 21.24 | 16.48 | 14.43 |
| Maoshan | 18.36 | 14.80 | 12.77 |
| Maoshan | 19.05 | 16.84 | 14.41 |
| Maoshan | 19.62 | 15.65 | 13.98 |
| Maoshan | 19.73 | 14.63 | 14.28 |
| Maoshan | 21.88 | 17.85 | 14.69 |
| Maoshan | 21.68 | 15.35 | 13.12 |
| Maoshan | 25.11 | 18.01 | 14.03 |
| Maoshan | 19.23 | 15.55 | 14.30 |
| Maoshan | 21.14 | 14.98 | 13.95 |
| Maoshan | 22.52 | 17.36 | 13.82 |
| Maoshan | 22.49 | 16.53 | 13.54 |
| Maoshan | 19.02 | 15.38 | 14.15 |
| Maoshan | 18.23 | 14.23 | 12.70 |
| Maoshan | 29.42 | 21.27 | 16.37 |
| Maoshan | 26.72 | 20.53 | 16.55 |
| Maoshan | 25.09 | 18.63 | 15.00 |
| Maoshan | 25.42 | 19.18 | 14.32 |
| Maoshan | 26.16 | 19.99 | 16.76 |
| Maoshan | 25.61 | 19.50 | 14.44 |
| Maoshan | 22.96 | 17.68 | 16.04 |
| Maoshan | 21.20 | 16.72 | 13.74 |
| Maoshan | 22.37 | 16.34 | 12.34 |
| Maoshan | 20.89 | 18.59 | 14.88 |
| Maoshan | 20.63 | 17.23 | 15.29 |
| Maoshan | 23.43 | 17.58 | 15.07 |
| Maoshan | 20.56 | 17.22 | 14.65 |
| Maoshan | 23.00 | 18.09 | 15.68 |
| Maoshan | 19.92 | 17.57 | 15.18 |
| Maoshan | 22.52 | 15.71 | 12.66 |
| Maoshan | 21.11 | 17.53 | 16.70 |
| Maoshan | 25.81 | 17.46 | 14.10 |
| Maoshan | 24.21 | 16.08 | 13.89 |
| Maoshan | 22.69 | 19.23 | 15.71 |
| Maoshan | 19.19 | 17.10 | 14.34 |
| Maoshan | 23.09 | 16.30 | 15.58 |
| Maoshan | 20.91 | 19.59 | 19.31 |
| Maoshan | 22.72 | 19.55 | 15.82 |
| Maoshan | 24.20 | 18.37 | 16.32 |
| Maoshan | 21.42 | 15.53 | 12.51 |
| Maoshan | 20.99 | 14.73 | 12.19 |
| Maoshan | 20.33 | 16.65 | 14.41 |
| Maoshan | 21.41 | 16.20 | 14.66 |
| Maoshan | 21.28 | 14.57 | 12.86 |
| Maoshan | 21.04 | 15.56 | 13.28 |
| Maoshan | 21.13 | 16.59 | 14.60 |
| Maoshan | 21.62 | 17.28 | 15.82 |
| Maoshan | 22.38 | 18.31 | 14.48 |
| Maoshan | 21.05 | 15.82 | 12.31 |
| Maoshan | 23.90 | 19.98 | 19.11 |
| Maoshan | 20.60 | 17.14 | 14.78 |
| Maoshan | 21.89 | 17.11 | 14.50 |
| Maoshan | 23.52 | 18.39 | 14.97 |
| Maoshan | 23.38 | 16.39 | 13.97 |
| Maoshan | 23.61 | 16.13 | 13.23 |
| Maoshan | 22.14 | 16.51 | 14.77 |
| Maoshan | 20.63 | 17.20 | 14.35 |
| Maoshan | 17.88 | 15.84 | 13.58 |
| Maoshan | 20.28 | 17.21 | 16.33 |
| Maoshan | 15.78 | 14.22 | 14.05 |
| Maoshan | 19.66 | 15.37 | 14.16 |
| Bianjiashan | 18.56 | 16.15 | 14.61 |
| Bianjiashan | 23.82 | 17.38 | 13.55 |
| Bianjiashan | 22.85 | 16.43 | 12.87 |
| Bianjiashan | 19.29 | 16.25 | 13.86 |
| Bianjiashan | 20.97 | 15.25 | 12.50 |
| Bianjiashan | 18.32 | 18.02 | 13.46 |
| Bianjiashan | 22.61 | 16.07 | 14.01 |
| Bianjiashan | 18.62 | 17.43 | 15.87 |
| Bianjiashan | 19.05 | 16.18 | 14.12 |
| Bianjiashan | 17.26 | 14.72 | 14.65 |
| Bianjiashan | 17.59 | 14.95 | 12.63 |
| Bianjiashan | 18.16 | 13.26 | 11.65 |
| Bianjiashan | 17.88 | 13.55 | 11.80 |
| Bianjiashan | 17.14 | 13.80 | 10.90 |
| Bianjiashan | 17.81 | 14.99 | 14.31 |
| Bianjiashan | 22.27 | 16.18 | 13.67 |
| Bianjiashan | 20.63 | 16.03 | 13.90 |
| Bianjiashan | 21.42 | 16.81 | 13.68 |
| Bianjiashan | 25.49 | 17.69 | 13.67 |
| Bianjiashan | 20.92 | 15.83 | - |
| Bianjiashan | 19.14 | 13.87 | - |
| Bianjiashan | 19.98 | 13.50 | - |
| Bianjiashan | 20.89 | 14.49 | - |
| Qianshanyang | 29.96 | 21.82 | 16.60 |
| Qianshanyang | 28.48 | 19.50 | 15.63 |
| Qianshanyang | 24.55 | 18.53 | 14.92 |
| Qianshanyang | 24.08 | 17.38 | 14.30 |
| Qianshanyang | 25.33 | 18.68 | 15.36 |
| Qianshanyang | 25.50 | 17.60 | - |
| Qianshanyang | 25.45 | 18.00 | - |
| Ikiriki | 21.50 | 17.10 | 13.50 |
| Ikiriki | 23.20 | 17.50 | 12.80 |
| Ikiriki | 21.70 | 17.50 | 13.00 |
| Ikiriki | 20.70 | 16.80 | - |
| Ikiriki | 25.10 | 20.20 | 15.90 |
| Ikiriki | 18.90 | 16.30 | 12.90 |
| Ikiriki | 22.60 | 19.60 | 14.40 |
| Ikiriki | 22.60 | 17.70 | 15.60 |
